# Posttranscriptional upregulation of HER3 by HER2 mRNA induces trastuzumab resistance in breast cancer

**DOI:** 10.1186/s12943-018-0862-5

**Published:** 2018-08-02

**Authors:** Xin Li, Yuxiu Xu, Yun Ding, Changfei Li, Hong Zhao, Jiandong Wang, Songdong Meng

**Affiliations:** 1 CAS Key Laboratory of Pathogenic Microbiology and Immunology, Institute of Microbiology, Chinese Academy of Sciences (CAS), Beijing, People’s Republic of China; 2 Cancer Institute and Hospital, Chinese Academy of Medical Sciences, Beijing, People’s Republic of China; 3 The General Hospital of People’s Liberation Army, Beijing, People’s Republic of China; 4 University of Chinese Academy of Sciences, Beijing, People’s Republic of China

**Keywords:** HER2, HER3, miR-125a/b, Trastuzumab resistance, Breast cancer

## Abstract

**Background:**

HER2 gene amplification generates an enormous number of HER2 transcripts, but the global effects on endogenous miRNA targets including HER family members in breast cancer are unexplored.

**Methods:**

We generated a HER2–3’UTR expressing vector to test the tumor-promoting properties in HER2 low expressing T47D and MCF7 cells. Through microarray analysis and real-time PCR analysis we identified genes that were regulated by HER2–3’UTR. Positive and negative manipulation of miRNA expression, response element mutational studies and transcript reporter assays were performed to explore the mechanism of competitive sequestration of miR125a/miRNA125b by HER2 3’UTR.

To investigate if trastuzumab-induced upregulation of HER3 is also mediated through miRNA de-repression, we used the CRISPR/cas9 to mutate the endogenous HER2 mRNA in HER2 over-expressing Au565 cells. Finally, we looked at cohorts of breast cancer samples of our own and the TCGA to show if HER2 and HER3 mRNAs correlate with each other.

**Results:**

The HER2 3’UTR pronouncedly promoted cell proliferation, colony formation, and breast tumor growth. High-throughput sequencing revealed a significant increase in HER3 mRNA and protein levels by the HER2 3’untranslated region (3’UTR). The HER2 3’UTR harboring a shared miR-125a/b response element induced miR-125a/b sequestration and thus resulted in HER3 mRNA derepression. Trastuzumab treatment upregulated HER3 via elevated HER2 mRNA expression, leading to trastuzumab resistance. Depletion of miR-125a/b enhanced the antitumor activity of trastuzumab. Microarray data from HER2-overexpressing primary breast cancer showed significant elevation of mRNAs for predicted miR-125a/b targets compared to non-targets.

**Conclusions:**

These results suggest that HER2 3’UTR-mediated HER3 upregulation is involved in breast cell transformation, increased tumor growth, and resistance to anti-HER2 therapy. The combinatorial targeting of HER3 mRNA or miR-125a/b may offer an effective tool for breast cancer therapy.

**Electronic supplementary material:**

The online version of this article (10.1186/s12943-018-0862-5) contains supplementary material, which is available to authorized users.

## Background

The HER-2/neu oncogene is amplified two-fold to > 20-fold in approximately 25% of breast cancers. Overexpression or gene amplification of HER2 is associated with poor prognosis and an aggressive course of the disease, such as oncogenic transformation, tumorigenesis, and metastasis [1]. HER2 belongs to the Type I receptor tyrosine kinase family, which includes four family members: EGFR (HER1), HER2 (neu or ERBB2), HER3, and HER4 [2]. In spite of possessing no known ligand, HER2 is the preferred heterodimerization partner within the family and can form heterodimers with HER1 and HER3, leading to phosphorylation of tyrosine residues within the cytoplasmic domain [3, 4]. Under certain circumstances, HER2 interacts with its binding partners, including MUC4, HSP90 or gp96, which may stabilize HER2 and make it endocytosis defective [5–8]. As a consequence HER2 heterodimerization with other HER members is resistant to downregulation, and induces a variety of signal transduction pathways, such as the PI3K/AKT, Ras/MAPK, and JAK/STAT pathways, leading to cell transformation and cancer [9, 10].

Due to its central role in aggressive tumor growth and metastases, HER2 serves as an ideal target for monoclonal antibody therapy, including the HER2 signaling inhibitor trastuzumab and pertuzumab, which can effectively treat tumors with HER2 gene amplification in 25% of patients as monotherapy and 50% when given with chemotherapy [11]. The application of HER2-targeted therapy dramatically changes the clinical outcome for HER2-positive breast cancer patients, even providing a superior prognosis compared to HER2-negative cases. However, acquired and inherent resistance to anti-HER2 therapy in these patients poses a serious challenge, and a better knowledge of the underlying mechanisms of sensitivity to anti-HER2 therapies is of extreme importance for the development of effective strategies to overcome resistance [12].

HER3 is overexpressed in 10–30% of breast cancer and is also associated with poor prognosis and worse survival [13]. The most important and well-understood signaling activity of HER3 is its unique and potent ability to activate downstream PI3K and AKT pathway signaling, which subsequently controls many biological processes critical for tumorigenesis, including translation, survival, anti-apoptosis, metabolic regulation, and cell cycle control [14]. In addition, HER3 is frequently co-expressed with HER2 in breast cancer, and high levels of HER2/HER3 dimerization are associated with poor survival prognosis in HER2-overexpressing breast cancer [15]. Of note, inhibition of HER2 or the PI3K-AKT pathway in HER2-overexpressing cells is followed by feedback upregulation of HER3, leading to attenuation of the response to inhibition [16, 17]. In HER2-positive metastatic breast cancer, high HER3 expression is linked to poor survival prognosis after anti-HER2 treatment [18, 19]. All of these studies indicate that there exists crosstalk and co-operativity between HER2 and HER3 expression, and thus, simultaneously targeting HER2 and HER3 may provide a more efficient therapy for breast cancer.

MicroRNAs (miRNAs) are a large family of small noncoding RNA molecules of approximately 22 nt in length that inhibit target gene expression by affecting mRNA stability or/and translational efficacy [20]. Mature miRNA duplexes are loaded onto the RNA-induced silencing complex (RISC), which contains a member of the RNA binding protein Argonaute family (Ago). They then pair with target sites (miRNA response elements, MREs) within the 3’untranslated regions (3’UTRs) of mRNAs to direct posttranscriptional downregulation [21]. Long noncoding RNAs (lncRNAs), pseudogenes, and even viral RNAs have been demonstrated to function as competitive endogenous RNAs (ceRNAs) that elevate the expression of the corresponding protein-coding genes with shared miRNA binding sites via competing with miRNA binding and derepressing the expression of these genes [22–24]. The relative abundance of ceRNAs vs. corresponding RNAs, levels of common miRNAs, and the number of MREs may all contribute to ceRNA interactions, according to a mathematical mass-action model for ceRNA networks [25]. The mechanism is of particular relevance to HER2-positive breast cancer, where amplification of the HER2 gene leads to an enormous number of HER2 mRNA transcripts [26].

Considering that HER2 gene amplification generates highly redundant mRNAs that harbor multiple miRNA binding sites, we speculate that HER2 mRNA acts as ceRNA and can sequester endogenous miRNAs within HER2 positive breast cancer cells, thus cross-regulating the stability and translational efficiency of other host mRNAs with shared miRNA response elements. In this study, we investigated the role of HER2 mRNA in breast cancer by analyzing potential HER2 mRNA-regulated miRNAs and the corresponding mRNA profiles. We further explored the impact of the interactions between HER2 and its corresponding mRNA on breast cancer growth and tumorigenesis. Our results provide valuable insights into the functional implications of HER2 mRNAs in anti-HER2 resistance.

## Methods

### Cell line

Human breast cancer cell lines AU565, BT474, T47D, and MCF7 and the human kidney 293 T cell line were obtained from Cell Resource Center, IBMS, CAMS/PUMC, China. Cell lines were passaged for < 6 months after receipt. Cell lines which were passed for > 6 months were identified by STR Classification. All cell lines were regularly tested negative for Mycoplasma. BT474.TtzmR and AU565.TtzmR sublines with acquired trastuzumab resistance were generated by continuous exposure of parental cells to increasing doses of trastuzumab (up to 10 μg/ml) for more than 6 months.

### sgRNA-CRISPR/Cas9 system design and construction

Potential target sites were predicted using “crispr.mit.edu” in the human genome, and two to three target sequences with low predicted scores for off-targets were chosen. Two complementary 20-bp oligonucleotides were annealed and cloned this into *Bbs*I-digested pSpCas9(BB)-2A-Puro (PX459). Then, cells were transfected with CRISPR/Cas9 sgRNA. Transfected cells were treated with puromycin at a concentration of 1 μg/ml. Surviving cells were sorted into 96-well plates by FACS. The genomic region encompassing the CRISPR/Cas9 target site was amplified and sequenced.

### Antibodies and reagents

Trastuzumab (Herceptin) was purchased from a pharmacy. PE-anti-HER2, APC-anti-HER3 antibody was from BioLegend (San Diego, California). Ago2-antibody was purchased from Abcam. All other antibodies were purchased from Cell Signaling Technology. The chemically synthesized specific siRNA, miRNA mimics, miRNA inhibitors, and non-specific control, as well as cholesterol-conjugated siRNA and miRNA mimics and control mimics, were purchased from RiboBio Co., Ltd. (Guangzhou, China).

### Real-time PCR

Total RNA was extracted with Trizol Reagent and quantified by real-time PCR using the SYBR Green Premix Reagent (Takara Bio Inc., Shiga, Japan) with an internal control for normalization.

### TaqMan miRNA analysis

Real-time PCR analysis for miR-125a/b was performed using a TaqMan miRNA Kit (Applied Biosystems). The U6 endogenous control was used for normalization. Relative expression was quantified using the comparative threshold cycle (Ct) method.

### Luciferase assay

To validate miRNA targeting, the 3’UTRs of HER2 and HER3 were cloned into the pGL3 firefly luciferase reporter plasmid. Cells were transfected in 48-well plates with 20 ng of pGL3 reporter, 2 ng of pRL-TK as the control, and 100 nM miRNA mimic. Firefly luciferase and Renilla luciferase activities were measured consecutively with the dual luciferase reporter system (Promega), and the firefly luciferase activity was normalized to that of Renilla luciferase after 48 h. To test the ceRNA activity of the HER2 3’UTR, 5× 10^4^ 293 T cells were transfected in 48-well plates with 20 ng of pGL3-HER3 3’UTR and 250 ng of pCDNA3.1-HER2 3’UTR or pCDNA3.1 as a control, as well as 1–10 nM miRNA mimic. Firefly luciferase and Renilla luciferase activities were measured consecutively with the dual luciferase reporter system (Promega), and the firefly luciferase activity was normalized to that of Renilla luciferase after 48 h.

### Transcriptional profiling by microarray

Comparative microarray analysis of mRNAs from T47D cells transfected with pCDNA3.1-HER2 3’UTR and pCDNA3.1 as a control was performed on an Agilent Whole Human Genome Microarray at the Shanghai Biotechnology Corporation according to manufacturer’s instructions.

### RNA immunoprecipitation

Cells were washed and lysed. Ago2-antibody or control IgG was incubated with ProteinG Sepharose beads (GE Healthcare). The beads were pelleted and washed and then subsequently incubated with cell lysate. After incubation, the beads were washed. RNA was isolated from the immunoprecipitated pellet by adding Trizol reagent. Total RNA was used for reverse transcription and real-time PCR analysis. The following primers were used: Ago-HER2 forward, 5’-AGCCGCGAGCACCCAAGT-3′ and reverse, 5′-TTGGTG GGCAGGTAGGTGAGTT’; and Ago-HER3 forward, 5’-GGGTTAGAGGAAGAGGATGTCAAC-3′ and reverse, 5′- GGGAGGAGGGAGTACCTTTGAG’.

### Transcript copy-number analysis

RNA was extracted from 1 × 10^5^ cells using Trizol Reagent. Absolute quantification of total HER2 and HER3 mRNAs and miR-125a/b was performed by real-time PCR. For a standardized evaluation, threshold cycle (CT) values were compared to a 10-fold serial dilution of either in vitro-transcribed HER2 or HER3 mRNAs or synthetic miR-125a/b mimics (RiboBio Co., Ltd). Then, the copies of transcript per cell were calculated with standard stoichiometric methods.

### Animal studies

Tumors were established by orthotopic injection of 1 × 10^7^ cells/mouse in 200 μl of PBS into the flanks of six-week-old female nude mice. The mice were randomly divided into groups at 15 d after inoculation. Tumor growth was measured every 3 days. Trastuzumab (10 mg/kg) was given intraperitoneally (i.p.) once a week. For delivery of cholesterol-conjugated siRNA, 10 nM siRNA in 0.1 ml PBS was locally injected into the tumor mass every 3 days for 2 weeks. Tumor growth was measured twice weekly, and the volume of the tumors was calculated as volume = length× width^2^ / 2.

### Immunohistochemistry analysis

Tissues were fixed in 4% paraformaldehyde overnight and embedded in paraffin according to standard procedures. Tissue sections were stained with the following procedures. Briefly, after deparaffinization and rehydration, antigen retrieval was performed using antigen retrieval butter in an autoclave at 121 °C for 100 s. Slides were then incubated with primary antibodies at room temperature for 40 min. Slides were washed with PBS and stained with fluorescence-conjugated secondary antibodies. Images were acquired using a Leica TCS SP8 confocal laser-scanning microscope (Leica Microsystems, Heidelberg, Germany).

### Breast primary tumor cells isolation and transfection

Informed written consents were obtained from breast cancer patients according to the General Hospital of People’s Liberation Army. Tumor biopsy was washed with PBS containing 100 U/ml penicillin and 100 μg/ml streptomycin, and was cut up into small pieces. Small tissues were minced in RPMI1640 medium. To obtain single cell suspensions, samples were treated with 1 mg/ml collagenase type IV and 1 mg/ml hyaluronidase followed by digestion with trypsin-EDTA at 37 °C for about 4 h with periodic agitation. After digestion, cells were centrifuged and cultured in RPMI1640. Fibroblast like elongated cells were visible firstly. Later, epithelial like cells started to make colonies in dome like shapes. These cell colonies were isolated and transferred to cell plates. These cells were passaged and expanded for transfection experiments.

Transfections were carried out using electroporation with Bio-rad transfection system according to the manufacturer’s instructions. Briefly, 1 × 10^6^ cells were transfected with 10 μg plasmids using a BioRad Gene Pulser II at 250 V and 950 μF. Each treatment was performed for at least three times.

### Expression data from the cancer genome atlas

The transcriptome expression profiles of breast cancer were downloaded from The Cancer Genome Atlas (TCGA) data portal (https://cancergenome.nih.gov). In this study, the transcriptome profiles of 887 cases and 101 HER2-positive breast tumors were included in the coexpression analysis. Level 3 Illumina miRNASeq was used to analyze miRNA expression. For the miRNASeq data, “reads_per_million_miRNA_mapped” values were used to calculate miRNAs.

### Statistical analyses

Data are expressed as the mean ± SD (standard deviation) from three independent experiments. The statistical significance between two and more than two groups was measured using the two-tailed Student’s t-test. *P* values < 0.05 were considered significant.

## Results

### The HER2 3’UTR enhances breast cancer cell malignancy

**Fig. 1:**
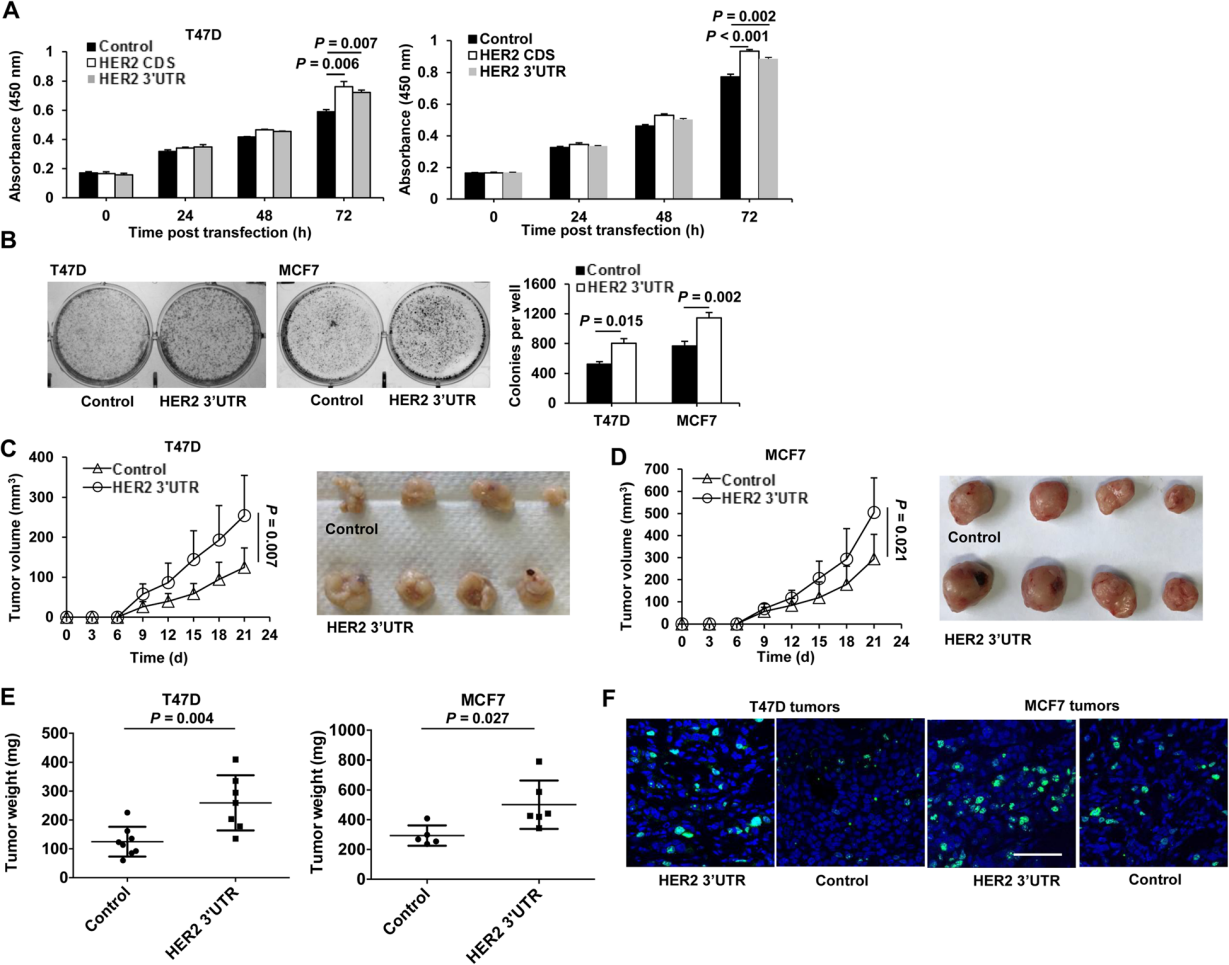
The HER2 3’UTR promotes cell proliferation, colony formation, and tumor growth of breast cancer. **a**, **b** Breast cancer cells were transfected with the HER2 3’UTR, HER2 CDS, or the empty vector as a control. Cell proliferationwas assessed by CCK-8 assays at the indicated times (**a**), and colony formation was determined 7 d post transfection (**b**). All quantitative data were generated from a minimum of three replicates. c-f T47D or MCF7 cells stably transfected with the HER2 3’UTR or control vector were s.c. injected into female BALB/c-nude mice. Tumor diameters were measured every 3 d for 21 d (**c**, **d**). Weight of xenograft tumors at 30 d post inoculation (**e**). Representative immunostaining of Ki67 in T47D and MCF7 xenograft tumors (**f**). Green, Ki67; Blue, DAPI. Scale bar, 40 μm. The results are presented as means±SD from five mice. Data are representative of two independent experiments

We first tested the effects of ectopic expression of the HER2 3’UTR in human breast cancer cells. Similar to cells transfected with the HER2 coding sequence (CDS), cells transfected with the HER2 3’UTR displayed increased cell proliferation compared to control vector-transfected cells (Fig. 1a). In addition, HER2 3’UTR-transfected cells formed more colonies than control cells (Fig. 1b). We further tested the tumor growth promoting ability of the HER2 3’UTR in vivo. As shown in Figs. 1c-e, HER2 3’UTR-transfected cells developed larger tumors compared to control cells (all *P* < 0.05 or 0.01). Tumor sections were stained for Ki67, a marker of cell proliferation. As shown in Fig. 1f, HER2 3’UTR-transfected tumors displayed a significantly higher level of Ki67-positive cells than that of the control cells.

### The HER2 3’UTR modulates HER3 expression

**Fig. 2:**
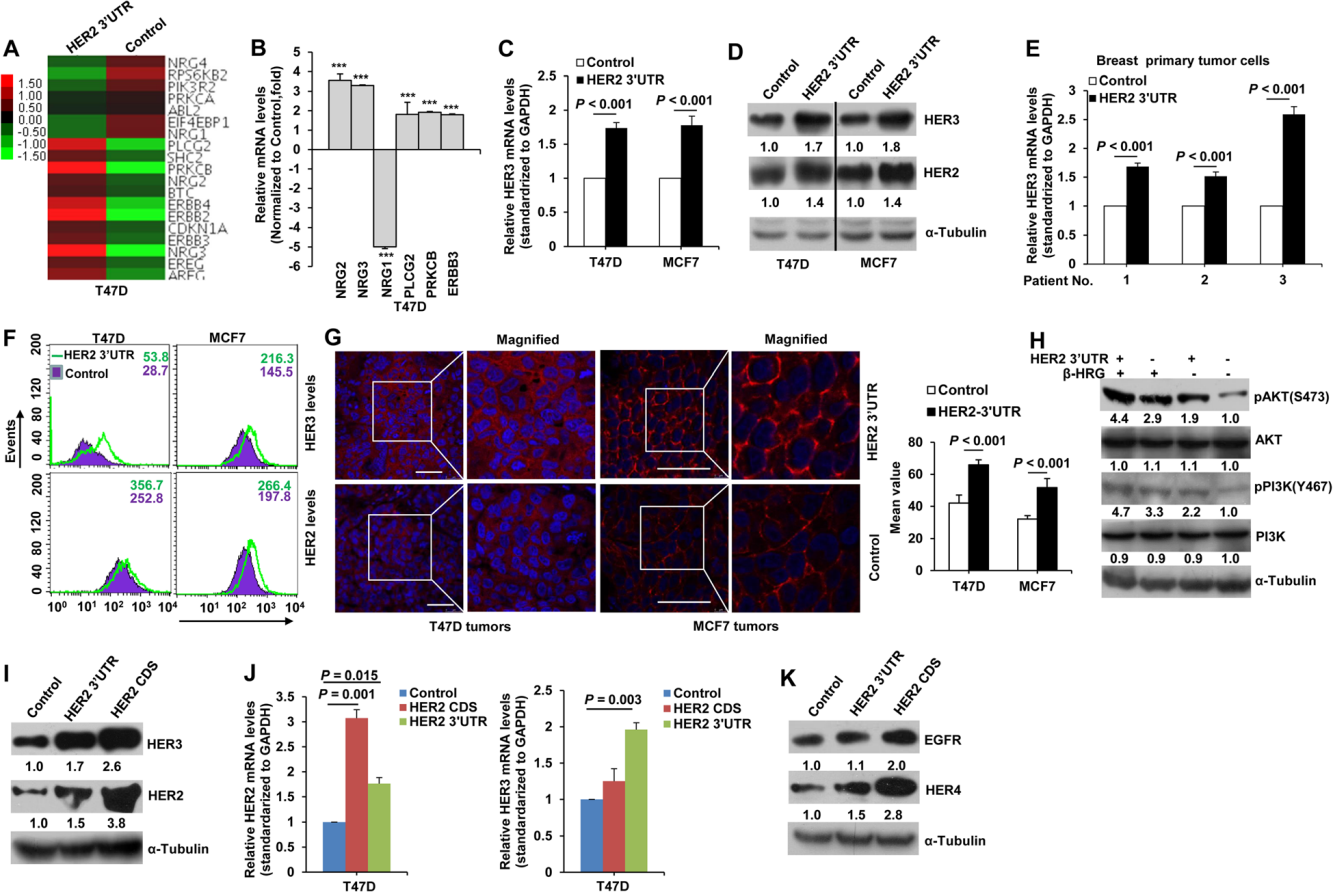
The HER2 3’UTR regulates HER3 expression. (A) Microarray heat map of differentially expressed genes in the ErbB pathway in T47D cells transfected with the HER2 3’UTR and control vector. See also Tables S1 and S2. (B) Real-time PCR analysis of gene expression in T47D cells as in (A). ^***^, *P* < 0.001 compared to the control. (C, D) Real-time PCR (C) and western blotting (D) analysis of HER3 mRNA and protein levels in T47D and MCF7 cells transfected with the HER2 3’UTR and control vector. (E) Real-time PCR analysis of HER3 expression in breast primary tumor cells transfected with the HER2 3’UTR and control vector. (F) FACS analysis of HER2 and HER3 expression in T47D and MCF7 cells stably transfected with the HER2 3’UTR or control vector. (G) Immunostaining of HER3 levels in T47D and MCF7 xenograft tumors. Red, HER3; Blue, DAPI. Scale bar, 40 μm. The mean values of HER3 fluorescence calculated and analyzed by Leica TCS SP8 Confocal platform are shown. (H) Western blot analysis of the PI3K/AKT pathway of T47D cells transfected with the HER2 3’UTR or control vector. (I, J, K) Western blot (I, K) and real-time PCR (J) analysis of EGFR, HER2, HER3 and HER4 protein and HER3 mRNA levels in T47D cells transfected with control vector, the HER2 3’UTR, or the HER2 CDS. All error bars represent the standard deviation. All quantitative data were generated from a minimum of three replicates

To explore HER2 3’UTR-dependent transcriptional programs, we analyzed gene-expression profiles of T47D cells transfected with the HER2 3’UTR. Compared to control cells, HER2 3’UTR-transfected cells showed upregulation of 4670 probes and downregulation of 4341 probes (Additional file 1: Table S1, log_2_(fold change) > 0.5). To define key pathways regulated by the HER2 3’UTR, gene-set enrichment analysis was performed. Among the top 50 enriched pathways, the Jak-STAT and ErbB signaling pathway were affected by the HER2 3’UTR (Additional file 2: Table S2). As the ErbB family plays a key role in tumor growth and development in breast cancer, we focused on the ErbB pathway in this study. As shown by microarray heat mapping, HER2 (ErbB2), HER3 (ErbB3), HER4 (ErbB4), NRG2, and NRG3 were up-regulated by expression of the HER2 3’UTR (Fig. 2a). The microarray results were further confirmed by real-time PCR analysis (Fig. 2b).

HER3 was selected for further study as the HER2–HER3 heterodimer constitutes the most mitogenic receptor complex and the key oncogenic unit within the HER family [14]. As represented in Fig. 2c and d, a significant increase of HER3 mRNA and protein levels was observed in HER2 3’UTR-transfected cells. Similar results were observed in primary breast cancer cells (Fig. 2e). Cell surface HER3 levels were elevated by ~ 1.6-fold and 1.5-fold for T47D and MCF7, respectively, as detected by flow cytometry (Fig. 2f). Furthermore, increased HER3 protein levels were observed in xenograft tumors stably transfected with the HER2 3’UTR, as detected by immunostaining (Fig. 2g). The HER2 3’UTR enhanced activation of AKT and PI3K signaling downstream of HER2/HER3 heterodimers (Fig. 2h). We further compared the effects of the HER2 3’UTR and HER2 CDS on HER2/HER3 expression. As can be seen in Fig. 2i, both the HER2 3’UTR and CDS increased HER2 and HER3 protein levels. However, only the HER2 3’UTR elevated HER3 mRNA levels (Fig. 2j), indicating that the HER2 3’UTR-induced increase in HER3 protein levels may be due to its elevated mRNA levels. HER2 CDS increased HER3 protein levels likely by forming heterodimers and reducing HER3 endocytosis and subsequent degradation [27]. In contrast, no obvious change in EGFR levels was observed by the HER2 3’UTR (Fig. 2k). The HER-2 3’UTR also elevated HER4 protein levels possibly through ceRNA crosstalk as the HER2 and HER4 3’UTRs have shared miRNA binding sites (Additional file 3: Table S3). Altogether, these data suggested that there is cross-talk between HER3 mRNA and the HER2 3’UTR.

### The HER2 3’UTR promotes cell malignancy in a miR-125a/b response element-dependent manner

**Fig. 3:**
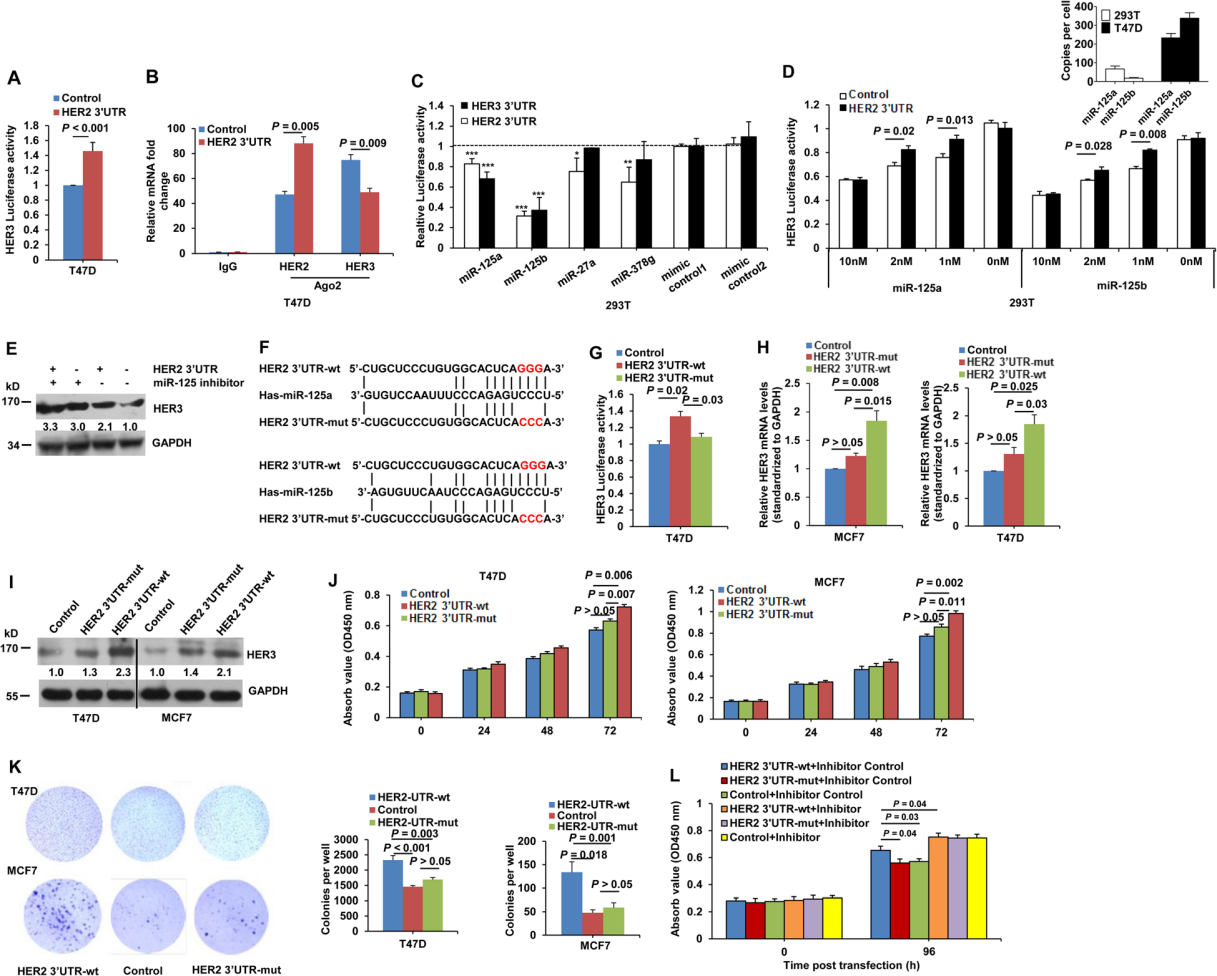
The HER2 3’UTR affects HER3 expression via miR125a/b. **a** HER3 3’UTR-luciferase reporter assay in T47D cells transfected with the HER2 3’UTR or control vector. **b** Lysates from T47D cells transfected with the HER2 3’UTR or control vector underwent immunoprecipitation with an anti-Ago2 antibody. Immunoprecipitated RNAs were recovered, and real-time PCR was performed using primers targeting the HER2 and HER3 3’UTRs. **c** 293 T cells were co-transfected with a luciferase reporter, the HER2 or HER3 3’UTR, the indicated miRNA mimic, or control mimic, respectively, and luciferase activities were measured using a dual-luciferase assay kit 48 h post transfection; see also Table S3. *P* < 0.05 (^*^) and *P* < 0.01 (^**^) compared to control transfections. **d** Lower panel, 293 T cells were co-transfected with HER3 3’UTR luciferase reporter constructs, the indicated dose of miR-125a or miR-125b mimics, and HER2 3’UTR or empty control plasmids. The luciferase activities were measured 48 h post transfection. Upper panel, copies-per-cell analysis of miR-125a/b in T47D and 293 T cells. **e** Western blotting of HER3 protein levels in T47D cells transfected with the HER2 3’UTR or control vector, along with miR-125a/b inhibitors or a randomized oligonucleotide (control). **f** Schematic representation of miR-125a/b response elements within the HER2 and HER3 3’UTR. Perfect matches are indicated by a line. Mutations were made in the seed region of the miR-125a/b binding sites. **g** Luciferase activity of the HER3 3’UTR reporter in T47D cells transfected with wild-type (wt) or mutant (mut) HER2 3’UTR or empty vector as a control. **h-k** MCF7 or T47D cells were transfected with wild-type or mutant HER2 3’UTR or empty vector as a control. Real-time PCR (**h**) and western blotting (**i**) were performed to determine HER3 mRNA and protein levels 24 and 48 h post transfection. Cell proliferation (**j**) and colony forming (**k**) were measured by CCK8 and clonogenic assays 72 h and 7 d post transfection, respectively. **l** Proliferation of T47D cells cotransfected with wild-type or mutant HER2 3’UTR or empty vector as a control, along with miR-125a/b inhibitors or a randomized oligonucleotide (control). All error bars represent the standard deviation. All quantitative data were generated from a minimum of three replicates

As shown in Fig. 3a, the HER2 3’UTR elevated the activity of a HER3 3’UTR luciferase reporter. In addition, HER3 mRNA was recruited to RISCs at much lower levels in HER2 3’UTR-transfected cells compared to control cells (Fig. 3b). MiRNA prediction indicated that 61 miRNAs have binding sites in both human HER2 and HER3 3’UTRs (Additional file 4: Table S4), among which miR-125-5p, miR-27a-5p, and miR-378 ranked in the top 100 in miRNA abundance in breast invasive carcinoma (TCGA). We then tested if these shared miRNAs are involved in regulation of HER3 expression via the HER2 3’UTR. miR-125a and miR-125b, which target the HER2 3’UTR at nucleotides 17–44 and 19–44, respectively [28, 29], significantly repressed HER2 and HER3 3’UTR luciferase reporters (Fig. 3c). Transfection of moderate amounts of miR-125a or miR-125b endowed the HER2 3’UTR with the capacity to regulate HER3 expression in miR-125a/b low expressing 293 T cells (Fig. 3d). No effect on HER3 luciferase reporter by HER2 3’UTR was observed in 293 T cells that were not transfected with miR-125a/b. This is due to low expression of miR-125a/b in 293 T cells, and a minimum amount of miR-125a/b is required for regulation of HER3 expression by HER2 3’UTR. In contrast, depletion of miR-125a and miR-125b with inhibitors blocked HER2 3’UTR-mediated elevation of HER3 expression in miR-125a/b-rich T47D cells (Fig. 3e).

To investigate the effect of miR-125a/b response elements on HER3 expression, mutations were introduced into the HER2 3’UTR within the miR-125a/b binding sites as shown in Fig. 3f. Up-regulation of the luciferase activity of the HER3 3’UTR (Fig. 3g), HER3 mRNA (Fig. 3h), and protein (Fig. 3I) levels was significantly attenuated by the mutated HER2 3’UTR, compared to wild type HER2 3’UTR. In addition, mutated HER2 3’UTR also significantly attenuated the ability to enhance cell proliferation (Fig. 3j) and colony formation (Fig. 3k). Finally, the effect of the HER2 3’UTR on cell proliferation was largely abolished by miR-125a and miR-125b depletion (Fig. 3l). Taken together, these results demonstrate that the miR-125a/b response element is essential for HER2 3’UTR regulation of HER3 and cell malignancy.

### Both the CDS and 3’UTR contribute to HER2 oncogenic potential

**Fig. 4:**
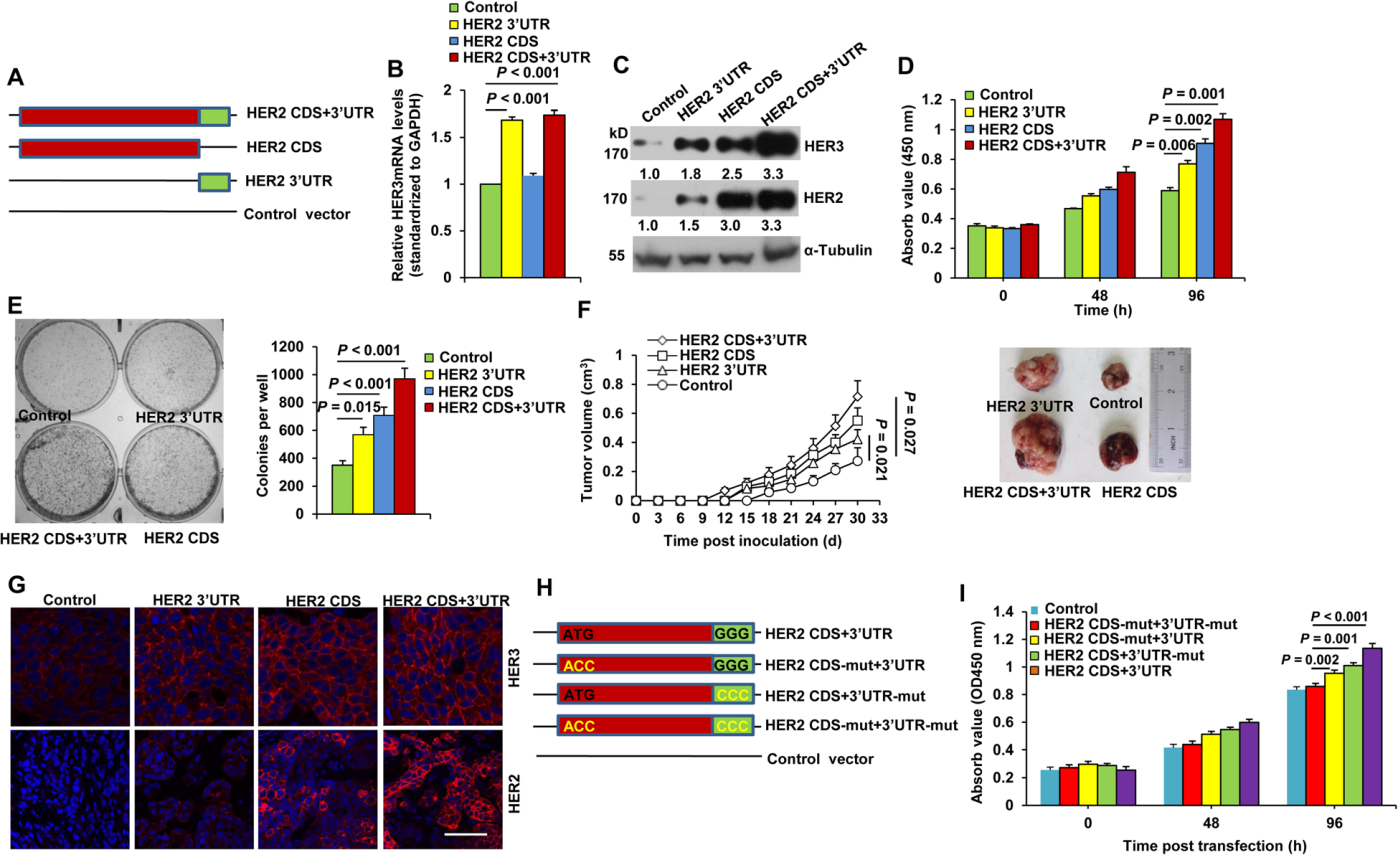
Both the HER2 CDS and 3’UTR possess oncogenic activity. **a** Schematic representation of the construction of HER2 mRNA elements. (B-E) T47D cells were transfected with the HER2 3’UTR, HER2 CDS, or full-length HER2. Real-time PCR (**b**) and western blotting (**c**) analyses were performed to determine HER3 mRNA and protein levels 24 or 48 h post transfection. Cell proliferation (**d**) and colony formation (**e**) were determined by CCK8 and clonogenic assays 96 h and 7 d posttransfection, respectively. All error bars represent the standard deviation. Quantitative data were generated from a minimum of three replicates. **f, g** T47D cells stably transfected with the HER2 3’UTR, HER2 CDS, or full-length HER2 were s.c. injected into female BALB/c-nude mice. Tumor diameters were measured every 3 d for 30 d (**f**, left). Representative tumor images at day 30 are shown (**f**, right). Immunostaining of HER3 and HER2 levels in T47D xenograft tumor sections (**g**). Red, HER2/HER3 as indicated; Blue, DAPI. Scale bar, 40 μm. The results are presented as means ± SD from five mice. **h, i** Schematic representation of the construction of HER2 mRNA elements. The start codon of the CDS and/or the seed region of the miR-125a/b binding sites were mutated as illustrated in yellow (**h**). T47D cells were transfected with plasmid constructs as in (H). Cell proliferation was determined by CCK8 assays 96 h post transfection. Error bars represent the standard deviation. Quantitative data were generated from a minimum of three replicates

We dissected the roles of the HER2 CDS and 3’UTR on HER2-mediated tumor growth. We constructed HER2 CDS, 3’UTR, and full-length HER2 (including both the CDS and 3’UTR) expression vector (Fig. 4a). The HER2 3’UTR and full-length HER2, but not the HER2 CDS, elicited a significant increase in HER3 mRNA levels (Fig. 4b), whereas all of the constructs increased HER3 protein levels, with full-length HER2 yielding the largest increase (Fig. 4c). Accordingly, transfection of the HER2 3’UTR, CDS, or the full-length construct all significantly increased cell growth and colony formation (Fig. 4d and e). Moreover, cells stably transfected with the HER2 3’UTR and CDS developed larger tumors compared to controls (Fig. 4f). Full-length HER2 led to the largest increase in cell proliferation, colony formation, and tumor growth. Increased HER3 expression was also observed in HER2 3’UTR-, CDS-, or full-length-overexpressing tumors (Fig. 4g). We further mutated the start codon of the HER2 CDS to prevent HER2 protein expression (Fig. 4h). As seen in Fig. 4i, transfection of this HER2 construct that ablates protein expression but preserves 3’UTR mRNA expression still stimulated cell proliferation.

### HER2 3’UTR-induced HER3 upregulation confers trastuzumab resistance

**Fig. 5:**
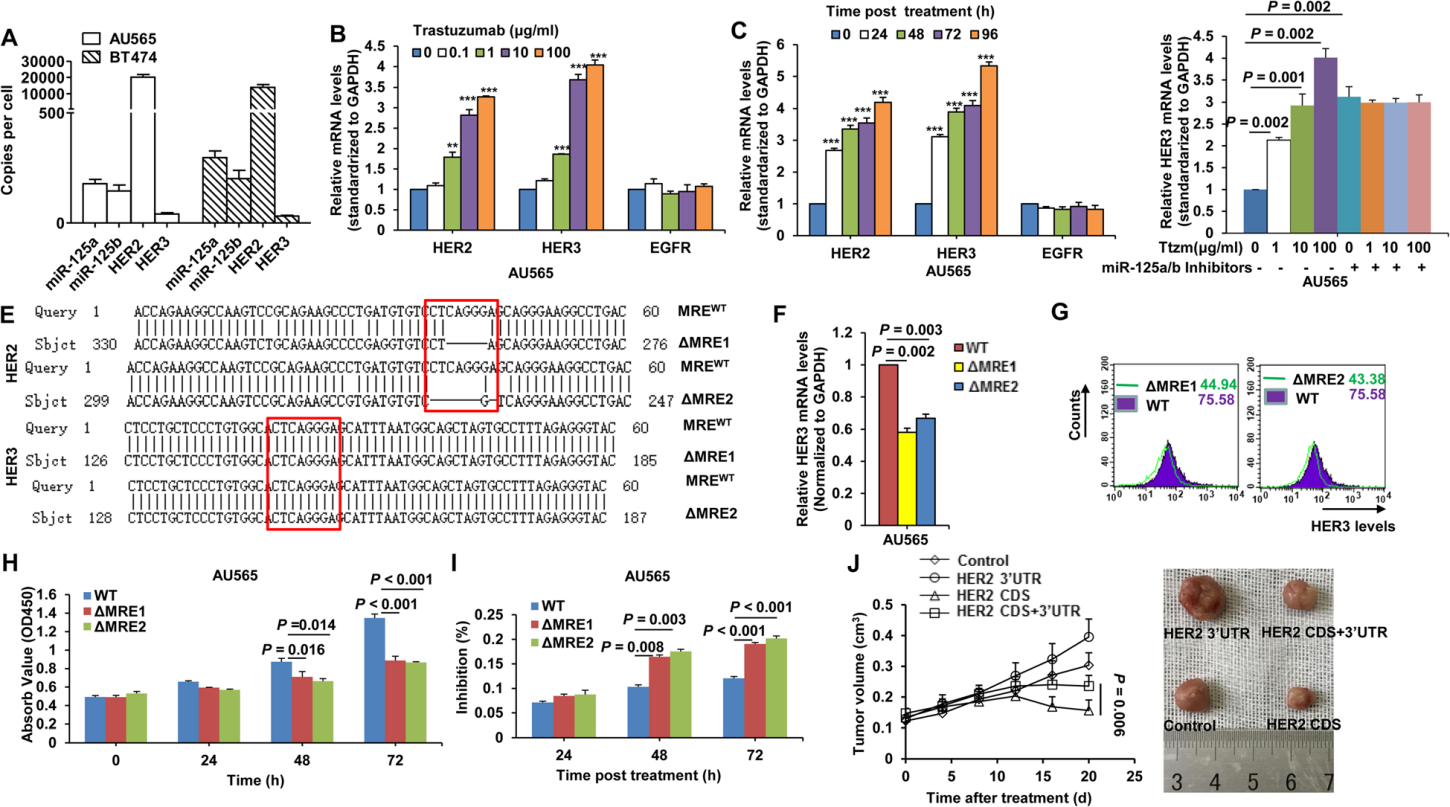
Effect of the HER2 3’UTR on trastuzumab resistance. **a** Copies-per-cell analysis of miR-125a/b, HER2, and HER3 mRNA in HER2-overexpressing cells. **b, c** Real-time PCR analysis of HER2 and HER3 mRNA levels in AU565 cells treated with the indicated concentration of trastuzumab (**b**) or 10 μg/ml trastuzumab at the indicated times (**c**). EGFR served as a negative control. ^**^, *P* < 0.01 compared to the control. ^***^, *P* < 0.001 compared to the control. **d** Real-time PCR analysis of HER3 mRNA levels in AU565 cells treated with the indicated concentration of trastuzumab with or without miR-125a/b inhibitors. **e** Sequencing of the HER2 and HER3 genes in wild type (MRE^WT^) and CRISPR/Cas9-mutated (ΔMRE) miR-125a/b response elements. Boxes in red indicate the seed region of the miR-125a/b binding sites. **f, g** Real-time PCR (**f**) and FACS (**g**) analyses of HER3 mRNA and protein levels in AU565 WT and ΔMRE cell lines. **h** Cell proliferation was determined by CCK8 assays. **i** AU565 WT and ΔMRE cells were treated with 10 μg/ml trastuzumab or control IgG at the indicated times. The inhibition rate of cell proliferation was calculated as: Inhibition rate (%) = [(cell proliferation without trastuzumab–cell proliferation with trastuzumab)/cell proliferation without trastuzumab] × 100%. **j** T47D cells stably transfected with the HER2 3’UTR, HER2 CDS, full-length HER2, or control vector were s.c. injected into female BALB/c-nude mice. When the xenograft tumors grew to a volume of 0.1 cm^3^, mice were treated with trastuzumab (10 mg/kg). Tumor diameters were measured every 5 d for 20 d (**j**, left). Representative tumor images at day 20 are shown (**j**, right). The results are presented as means ± SD from five mice

An important consideration in the HER2 mRNA-miR-125a/b-HER3 mRNA interaction is their absolute levels in cells, so we measured the copy numbers of their transcripts by real-time PCR calibrated with an internal standard curve of a HER2 or HER3 expression vector or synthetic miR-125a/b mimics. As shown in Fig. 5a, there were ~ 10,000–20,000 HER2 mRNA transcripts per cell in HER2 over-expressing cells. miR-125a/b was expressed at ~ 100–200 molecules per cell. In contrast, HER3 mRNA was expressed at only ~ 30 molecules per cell. A low ratio of miR-125a/b:HER2 mRNAs and high ratios of miR-125a/b:HER3 and HER2:HER3 mRNAs were found in HER2 over-expressing cells, which further indicates that the interaction between HER2 and HER3 mRNAs occurs under such a circumstance. Of note, trastuzumab treatment led to upregulation of HER2 and HER3 mRNA levels in both dose-dependent and time-dependent manners (Fig. 5b-c). Trastuzumab had no obvious effect on miR-125a expression levels and only slightly decreased miR-125b levels (Additional file 5: Figure S1). Only a moderate decrease in HER3 protein levels was observed by transient treatment with trastuzumab (Additional file 6: Figure S2), which may be because the amount of increased HER3 by HER2 mRNAs could not totally compensate for the loss of HER3 by decreased HER2 protein. The enhancement of trastuzumab on HER3 mRNA expression was largely abrogated in cells treated with miR-125a and miR-125b inhibitors (Fig. 5d), validating that upregulation of HER3 mRNA by trastuzumab was dependent on miR125a/b.

AU565 cells were then treated with increasing doses of trastuzumab for more than 6 months to obtain a trastuzumab-resistant clone, TtzmR. Both HER2 and HER3 mRNA levels were increased in TtzmR cells compared to parental cells (Additional file 7: Figure S3A). In addition, a substantial downregulation of HER2 protein levels and an obvious upregulation of HER3 were observed in the trastuzumab-resistant cells (Additional file 7: Figure S3B, S3C). We then investigated whether combinatory treatment with HER3 siRNA and trastuzumab could be a useful strategy for overcoming trastuzumab resistance. As shown in Figures (Additional file 7: Figure S3D and S3E, knocking down HER3 significantly restored the sensitivity of the trastuzumab-resistant cells to trastuzumab treatment, both in vitro and in vivo. The efficiency of HER3 knock down by RNAi was determined by IF staining (Additional file 7: Figure S3F). To further determine the role of the HER2 3’UTR on trastuzumab resistance, we generated a mutant AU565 cell line lacking the seed sequence within the miR-125a/b responsive element (ΔMRE) by CRISPR/Cas9. Two clones were selected after sequencing (Fig. 5e). Compared to wild type cells, ΔMRE cells displayed decreased levels of HER3 mRNA (Fig. 5f) and protein (Fig. 5g), as well as decreased cell proliferation (Fig. 5h). In addition, greater inhibition by trastuzumab was observed in mutant cells (Fig. 5i). Finally, cells stably expressing the HER2 3’UTR displayed increased tumor resistance to trastuzumab treatment (Fig. 5j). Together, these data indicate that the HER2 3’UTR is involved in trastuzumab resistance in breast cancer.

### Correlation between HER2 and HER3 levels in primary breast cancer

**Fig. 6:**
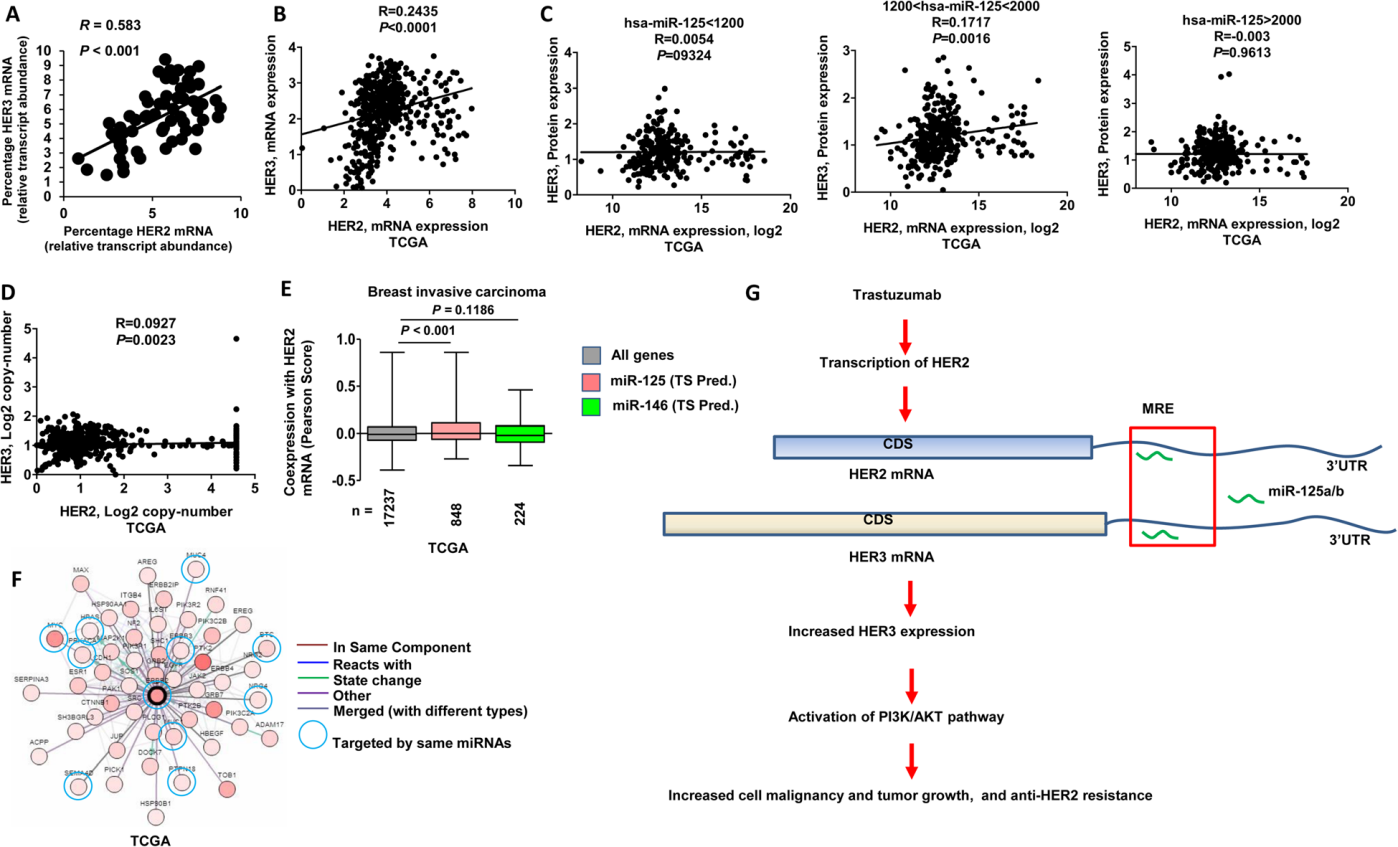
Correlation between HER2 and HER3 levels in human breast cancer. **a** Correlation of HER2 and HER3 mRNA expression in 80 primary human breast tumors. **b-d** Correlation of HER2 and HER3 mRNA (B) HER2 mRNA and HER3 protein levels **c**, HER2 and HER3gene copy-number **d** in breast invasive carcinoma (TCGA). **e** Meta-analysis of gene expression microarray data. miR-125a/b and miR-146a targets predicted by TargetScan (TS) were analyzed. The Pearson score of coexpression with HER2 mRNA of miR-125a/b targets was compared to miR-146a targets or all genes; the number of genes in each group is shown. **f** Meta-analysis of gene interaction with HER2. Genes targeted by the same miRNAs are circled. **g** A schematic model of HER2-HER3 mRNA cross-talk in breast tumor development and trastuzumab resistance

We further assessed HER2 and HER3 mRNA levels in breast cancer patients. Quantitative real-time PCR analysis revealed a significant positive correlation between HER2 and HER3 mRNA levels in 80 breast cancer tumors (Fig. 6a). Sequencing of the HER2 3’UTR showed that there was strict sequence conservation among tumors (data not shown). We next interrogated The Cancer Genome Atlas’s data for HER2 and HER3 expression. As shown in Fig. 6b, there is a significant correlation between HER2 and HER3 mRNA levels in breast cancer. Importantly, a positive correlation between HER2 mRNA and HER3 protein levels was observed in patients with medium amounts of miR-125a/b (Fig. 6c), which is consistent with the observation in cell experiments (Fig. 3d). However, no such correlation was seen between HER2 and HER3 gene copy numbers (Fig. 6d). There is no correlation between miR-125a/b and protein levels of HER1 and HER2 in HER2 positive patients (Additional file 8: Figure S4), which may be due to a high ratio of HER2 mRNAs:miR-125a/b. In addition, no correlation between miR-125a and HER3 protein and only a weak correlation between miR-125b and HER3 protein level were observed, indicating that highly abundant HER2 mRNAs may sequester most miR-125a/b. Taken together, these data suggest that HER2 mRNA upregulates HER3 expression through miR-125a/b in primary breast cancer. We further performed a meta-analysis using TCGA data. By comparing expression levels of predicted miR-125a/b targets to those of all genes in the microarray data, we found a significant elevation in predicted miR-125a/b targets compared to all genes or to non-miR-125a/b targets (Fig. 6e). Aside from HER3, HER2 mRNA may act as a sponge to absorb other shared miRNAs and up-regulate the corresponding oncogenes, such as MYC, mucin 1/4 (MUC1 and MUC4), semaphorin 4D (Sema4D), and HRAS (Fig. 6f).

## Discussion

HER2 heterodimerization with other HER members leads to the most potent of receptor combinations for causing continual downstream PI3K/Akt, Ras/MAPK, and JAK/STAT signaling, which drives oncogenic transformation and breast tumor growth. In the present study, we investigated whether highly expressed HER2 mRNAs in HER2 gene-amplified breast cancer could directly affect the expression of other HER members via shared MREs. We focused on HER3 because, as a specialized allosteric activator, it has a unique and potent ability to activate the downstream PI3K and AKT pathways. We showed herein that the HER2 3’UTR derepresses HER3 by sequestering cellular miR-125a/b in breast cancer cells and tumors. The tumor growth promotion effect of the HER2 3’UTR was nearly comparable to the HER2 CDS (Fig. 4). Our study establishes the HER2 3’UTR as a potent oncogenic transcript that elevates the expression of HER3 and activates downstream AKT and PI3K pathways in a MRE-dependent and coding-independent manner. These results are consistent with the observation of the positive correlation between HER2 and HER3 mRNA levels in breast cancer patients. Remarkably, we demonstrated that inhibition of HER2 with trastuzumab results in time- and dose-dependent upregulation of HER3 mRNA and protein by the HER2 3’UTR, which confers trastuzumab resistance. Our results may therefore provide further dissection of the impact of HER2 overexpression on both HER3 mRNA regulation and anti-HER2 resistance, as shown in Fig. 6g. In this model, HER2 mRNA-mediated HER3 up-regulation plays an important role in breast cell transformation, tumor growth, and resistance to anti-HER2 therapy. These data imply that targeting HER2/HER3 mRNAs may provide an effective strategy for the treatment of HER2-positive breast cancer.

In HER2-amplified cancer, activation of HER3 may occur through high-level expression of heterodimerization with HER2 [30]. HER3 is classified as a pseudokinase which lacks intrinsic kinase activity. HER2 has no known ligands but can dimerize with other HER family members, especially HER3. HER3 can be phosphorylated and activated by residual HER2 activity. Phosphorylated HER3 in turn activates PI3K via its six docking sites for the p85 adaptor subunit of PI3K. Due to HER3-mediated compensation, current clinical therapy against HER2 will not block PI3K pathway completely, resulting in unrestrained PI3K-AKT-mTOR signaling that is essential for tumorigenesis and drug resistance [14, 31, 32]. In breast carcinoma, HER3 levels can significantly increase due to overexpression instigated via gene amplification [33], transcription, and protein translation [15, 17, 34] or via enhanced molecular stability by dimerization [27]. miRNAs affect HER3 expression at the post-transcription level [29, 35–38]. Both HER2 and HER3 are validated targets of miR-125a and miR-125b [28].

HER2/HER3 co-overexpression is significantly associated with metastasis and shorter survival in breast cancer [13, 39]. All of these studies indicate that there may be crosstalk and cooperativity between HER2 and HER3. Our current work suggests that besides by HER2 protein heterodimerization, HER2 overexpression in breast cancer up-regulates HER3 via the HER2 3’UTR, which acts as a sponge to bind and sequester endogenous miR-125a/b. The HER2 3’UTR increases HER3 mRNA levels in a miR-125a/b response element- and miR-125a/b-dependent manner. HER2 3’UTR-induced miR-125a/b sequestration results in reduced Ago2 binding and HER3 mRNA derepression. In addition, a low ratio of miR-125a/b: HER2mRNAs and high ratios of miR-125a/b: HER3 mRNA and HER2:HER3 mRNAs were displayed in HER2 gene-amplified and protein-overexpressing breast cancer cells (Fig. 5a), which further indicates that the interaction between HER2 and HER3 mRNA may occur under such a circumstance. We consider that HER2 3’UTR preferentially regulates HER3 mRNA level but not vice versa, as the binding affinity between HER2 3’UTR and miR-125a/b was relatively higher than that between HER3 3’UTR and miR-125a/b by the maximum free energy predicted hybridization configurations (data not shown) and luciferase reporter assay (Fig. 3a and Additional file 9: Figure S5), and a high ratio of HER2:HER3 mRNAs were found in HER2 gene amplified cells (Fig. 5a). Besides HER3, expression levels of HER4 in HER2 3’UTR-transfected cells were also increased (Fig. 2a, k). MiRNA prediction indicated that 46 miRNAs have binding sites in both human HER2 and HER4 3’UTRs (Additional file 3: Table S3). These data suggested that HER2 may also regulate HER4 expression through ceRNA crosstalk.

Trastuzumab and pertuzumab, monoclonal antibodies targeting HER2 homodimerization and heterodimerization, as well as the HER2 tyrosine kinase inhibitor (TKI), display considerable clinical efficiency. However, the high prevalence of drug resistance after continuous treatment is a major concern [40, 41]. Several lines of evidence demonstrate that upregulation of HER3 is one of the main roads to resistance to anti-HER2 therapies [42, 43]. In current study we found that trastuzumab treatment upregulated HER3 expression via elevated HER2 3’UTR. Although anti-HER2 therapies lead to substantially decreased HER2 level, upregulated HER3 may form dimmers with other kinase including EGFR, FGFR, Met, Src that could also phosphorylate and activate HER3 [16, 44–46]. In addition, even the maximal doses of trastuzumab did not totally deplete HER2 expression (see Additional file 10: Figure S6), it is possible that the residual HER2 kinase activity was enough to partly maintain HER3 phosphorylation. All these could lead to resistance to HER2-targeted therapies. Breast cancer patients with high levels of HER2/HER3 dimerization have poor survival prognosis under treatment with adjuvant trastuzumab [15]. Moreover, a phase II clinical trial study shows that that low HER3 mRNA may represent a pertuzumab-sensitive phenotype in an enriched ovarian cancer patient [47]. Besides, elevated expression of HER3 and MUC4 and their interactions that possibly induced by increased phosphorylation of ERK and expression of PI3K and c-Myc were observed in HER2 knockdown pancreatic cancer cells, leading to increased cell proliferation, motility and tumorigenicity [48]. The feedback mechanisms of inhibition of HER2 or the PI3K/AKT pathway lead to a rebound in HER3 expression and signaling, which provides a rationale for the combination of targeted-therapies and drugs targeting HER2 and HER3. Indeed, dual targeting of HER2 and HER2/HER3 dimerization with trastuzumab and pertuzumab results in enhanced tumor inhibitory effects in mouse xenograft models, and significantly prolongs progression-free survival in HER2-overexpressing breast cancer [11, 49, 50].

Consistent with these previous studies, our findings show that continuous treatment with trastuzumab leads to a substantial down-regulation of HER2 protein levels but a compensatory upregulation of HER2 mRNA, thereby leading to increased HER3 mRNA levels through ceRNA crosstalk. Trastuzumab upregulated HER3 in a miR125a/b-dependent manner as elevated HER2 mRNA by trastuzumab treatment sequestered endogenous miR-125a/b by its 3’UTR and subsequently derepressed HER3 mRNA. Targeting of HER3 with siRNA and/or mutation of the miR-125a/b responsive element within the HER2 3’UTR sensitized HER2-overexpressing breast cancer cells and xenografts to trastuzumab. Our study therefore presents an effort to address the mechanism of regulation of HER3 mediated under anti-HER2 treatment, which further supports the notion that inhibition of HER3, especially targeting HER2 mRNA might provide a novel therapeutic approach for HER2-targeted therapies in breast cancer.

The sensitivity of breast cancer to trastuzumab is directly related to HER2 expression levels, and elevated HER3 is involved in trastuzumab resistance. Although transfection of HER2 CDS and full-length gene both led to increased HER3 expression, they highly elevated HER2 expression in the meantime (see Fig. 4c and g), transforming T47D cells into HER2-positive cells and therefore making these cells more sensitive to trastuzumab. In contrast, HER2 3’UTR only moderately increased HER2 level. This may explain why cells stably expressing the HER2 3’UTR displayed more resistance to trastuzumab treatment than those expressing HER2 CDS or full-length gene (see Fig. 5j). As trastuzumab-induced upregulation of HER3 mRNA was largely dependent on miR125a/b and the HER2 3’UTR (see Fig. 5), we believe that HER2 3’UTR-mediated HER3 upregulation plays an important role in the increased HER3 expression under trastuzumab treatment. In addition, it is worthwhile to fully dissect the cross-regulatory function of HER2 mRNAs on other pathways and their casual role in breast cancer development and drug resistance.

## Conclusion

Our study demonstrates that HER2 mRNAs posttranscriptionally up-regulate HER3 via the sequestration of miR-125a/b, contributing to enhanced breast cancer growth and acquired anti-HER2 resistance. Given that HER2 amplification in breast cancer usually generates highly redundant transcripts, our study therefore supports the notion that the inhibition of HER2 mRNA and/or miR-125a/b activity might provide a novel therapeutic approach for combined targeted drug administration.

## Additional files

Additional file 1: **Table S1.** Gene fold changes in T47D cells transfected with HER2–3’UTR or control vector. (XLSX 891 kb)

Additional file 2: **Table S2.** Gene-set enrichment analysis in T47D cells transfected with HER2–3’UTR or control vector. (XLSX 37 kb)

Additional file 3: **Table S3.** Shared miRNAs binding sites prediction in human HER2 and HER4 3’UTR. (XLSX 12 kb)

Additional file 4: **Table S4.** miRNAs binding sites prediction in human HER2 and HER3 3’UTR. (XLSX 26 kb)

Additional file 5: **Figure S1.** Effect of trastuzumab on miR-125a/b levels. Real-time PCR analysis of miR-125a and miR-125b levels in AU565 cells treated with 10 μg/ml trastuzumab at the indicated times. Data were generated from three replicates. (PDF 295 kb)

Additional file 6: **Figure S2.** Effect of trastuzumab on HER2 and HER3 levels. FACS analysis of HER2 and HER3 levels in AU565 cells treated with 10 μg/ml trastuzumab at the indicated times or the indicated concentrations of trastuzumab. (PDF 1027 kb)

Additional file 7: **Figure S3.** Combinatory treatment with HER3 siRNA and trastuzumab is useful for overcoming trastuzumab resistance. (A-C) Real-time PCR (A), western blotting (B), and FACS (C) analysis of HER2 and HER3 expression in AU565 parental and trastuzumab-resistant (TtzmR) cell lines. (D) Proliferation of AU565 parental and TtzmR cells treated with 10 μg/ml trastuzumab or control IgG, along with a cholesterol-conjugated siRNA targeting HER3 or a randomized oligonucleotide (control). All error bars represent the standard deviation. All quantitative data were generated from a minimum of three replicates. (E, F) AU565 TtzmR cells were s.c. injected into female BALB/c-nude mice. Mice were treated with cholesterol-conjugated HER3 siRNA or trastuzumab at days 0, 7, and 14. Representative in vivo luciferase images of mice at days 0, 10, and 21(E). The results are presented as means ± SD from five mice. Immunostaining of HER3 in xenograft tumor sections (F). Red, HER3; Blue, DAPI. Scale bar, 40 μm. (PDF 3149 kb)

Additional file 8: **Figure S4.** Correlation between miR-125a/b and EGFR family proteins in HER2 positive breast cancer patients. Correlation between miR-125a and miR-125b and EGFR family proteins (EGFR, HER2 and HER3) in HER2 positive breast cancer patients. (PDF 1450 kb)

Additional file 9: **Figure S5.** Reciprocal ceRNA activity between HER2 and HER3 3’UTR. HER2 3’UTR-luciferase reporter assay in T47D cells transfected with the HER3 3’UTR or control vector. (PDF 150 kb)

Additional file 10: **Figure S6.** Effect of trastuzumab on HER2 protein levels. Western blot analysis of HER2 levels in AU565 cells treated with the indicated concentrations of trastuzumab. Numbers below the blot indicates quantification shown on Western blot after normalization. (PDF 282 kb)

## Abbreviations

3’UTR: 3’untranslated regions
CDS: Coding sequence
ceRNA: competitive endogenous RNA
FACS: Fluorescence-activated cell sorting
HER2: Human epidermal growth factor receptor 2
HER3: Human epidermal growth factor receptor 3
miRNAs: microRNAs
MREs: iRNA response elements
RISC: RNA-induced silencing complex
TCGA: The Cancer Genome Atlas
TtzmR: Trastuzumab-resistant
