# Supplementary figures and images for: Posttranscriptional upregulation of HER3 by HER2 mRNA induces trastuzumab resistance in breast cancer
Source: Mol Cancer. 2018 Aug 2;17:113. doi: 10.1186/s12943-018-0862-5 (PMC6090962; doi:10.1186/s12943-018-0862-5)

# Figure S1

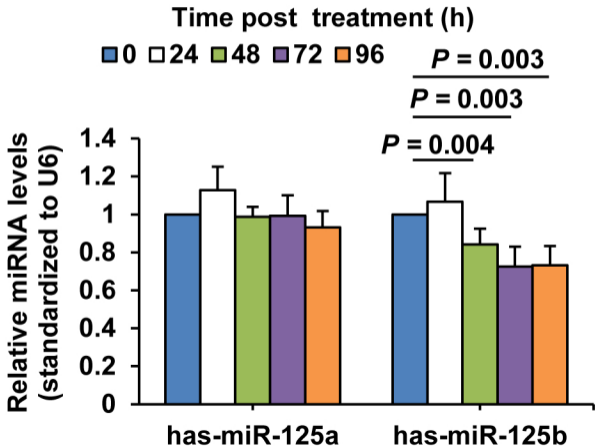

Supplement: Supplementary file 5 — Figure S1. Effect of trastuzumab on miR-125a/b levels. Real-time PCR analysis of miR-125a and miR-125b levels in AU565 cells treated with 10 μg/ml trastuzumab at the indicated times. Data were generated from three replicates. (PDF 295 kb) [file 12943_2018_862_MOESM5_ESM.pdf]

Figure S2

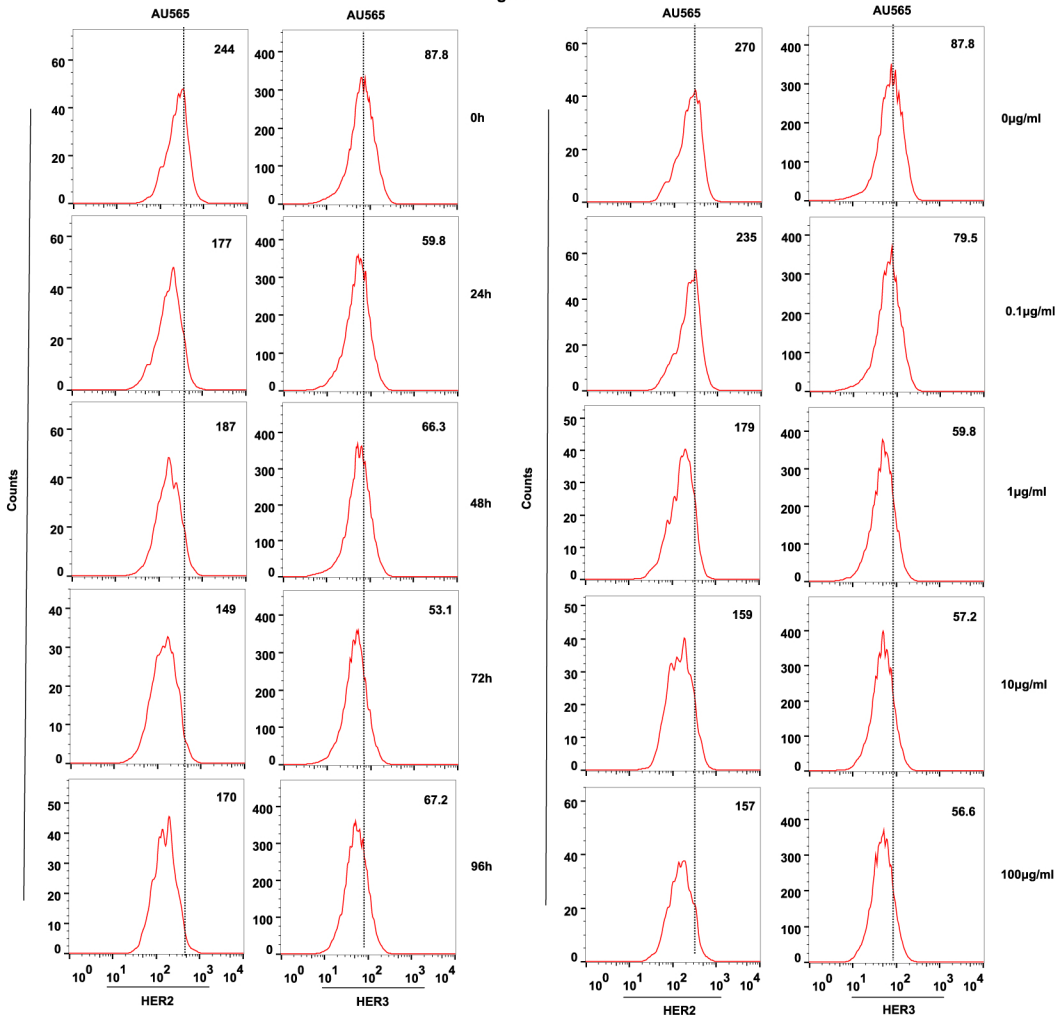

Supplement: Supplementary file 6 — Figure S2. Effect of trastuzumab on HER2 and HER3 levels. FACS analysis of HER2 and HER3 levels in AU565 cells treated with 10 μg/ml trastuzumab at the indicated times or the indicated concentrations of trastuzumab. (PDF 1027 kb) [file 12943_2018_862_MOESM6_ESM.pdf]

Figure S3

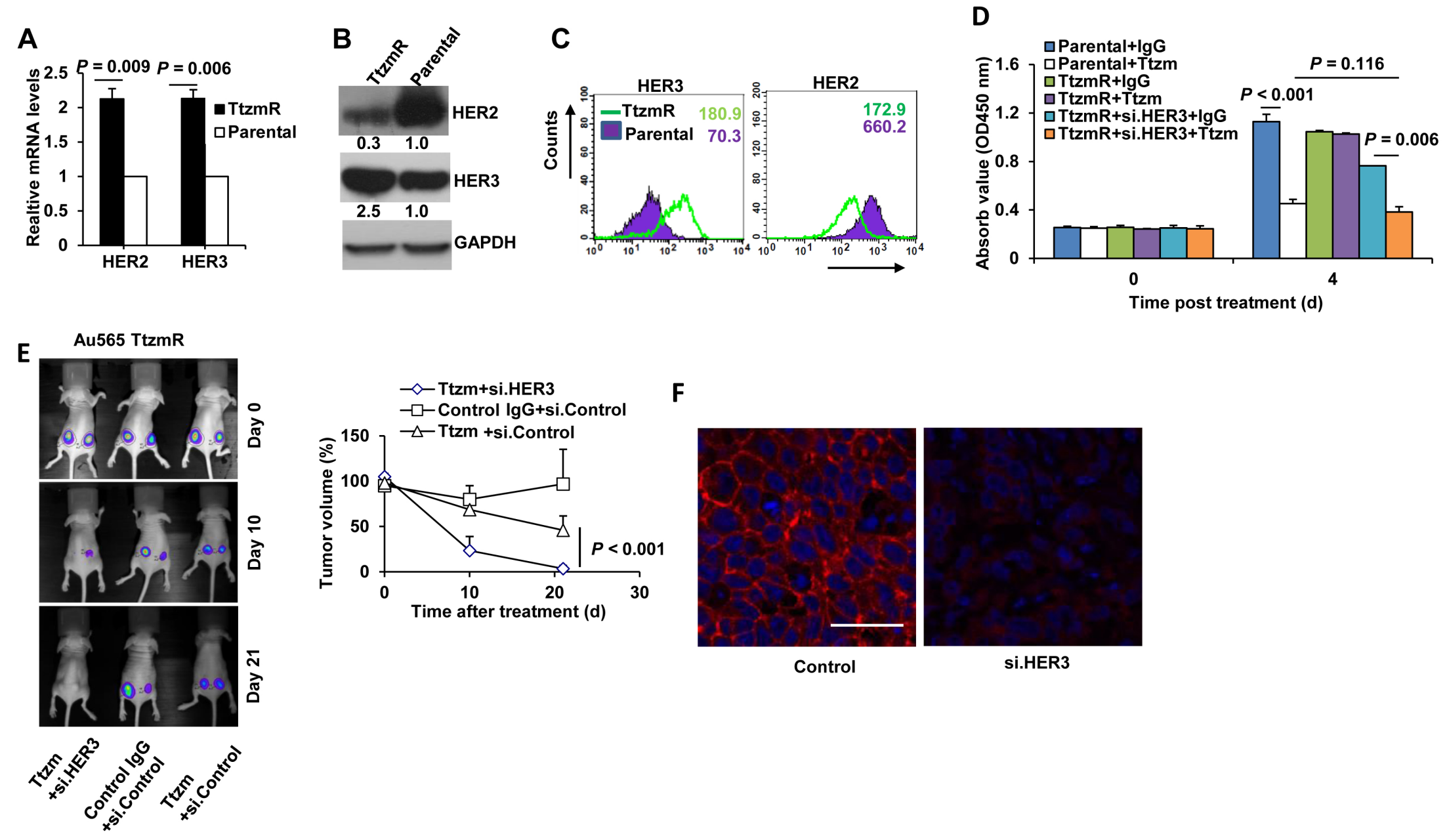

Supplement: Supplementary file 7 — Figure S3. Combinatory treatment with HER3 siRNA and trastuzumab is useful for overcoming trastuzumab resistance. (A-C) Real-time PCR (A), western blotting (B), and FACS (C) analysis of HER2 and HER3 expression in AU565 parental and trastuzumab-resistant (TtzmR) cell lines. (D) Proliferation of AU565 parental and TtzmR cells treated with 10 μg/ml trastuzumab or control IgG, along with a cholesterol-conjugated siRNA targeting HER3 or a randomized oligonucleotide (control). All error bars represent the standard deviation. All quantitative data were generated from a minimum of three replicates. (E, F) AU565 TtzmR cells were s.c. injected into female BALB/c-nude mice. Mice were treated with cholesterol-conjugated HER3 siRNA or trastuzumab at days 0, 7, and 14. Representative in vivo luciferase images of mice at days 0, 10, and 21(E). The results are presented as means ± SD from five mice. Immunostaining of HER3 in xenograft tumor sections (F). Red, HER3; Blue, DAPI. Scale bar, 40 μm. (PDF 3149 kb) [file 12943_2018_862_MOESM7_ESM.pdf]

Figure S4

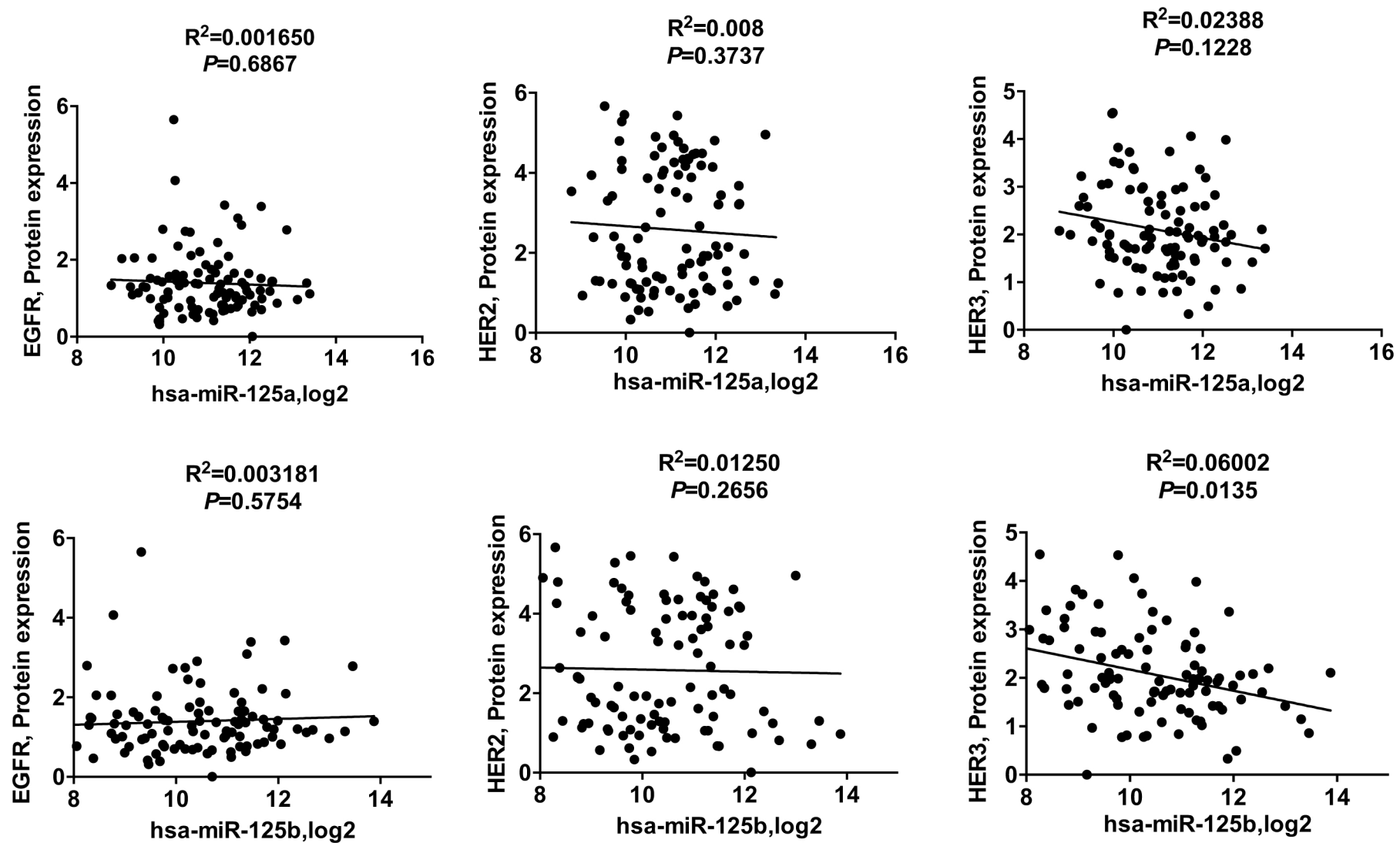

TCGA

Supplement: Supplementary file 8 — Figure S4. Correlation between miR-125a/b and EGFR family proteins in HER2 positive breast cancer patients. Correlation between miR-125a and miR-125b and EGFR family proteins (EGFR, HER2 and HER3) in HER2 positive breast cancer patients. (PDF 1450 kb) [file 12943_2018_862_MOESM8_ESM.pdf]

# Figure S5

■ Control

■ HER3 3'UTR

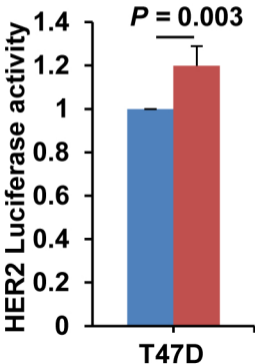

Supplement: Supplementary file 9 — Figure S5. Reciprocal ceRNA activity between HER2 and HER3 3’UTR. HER2 3’UTR-luciferase reporter assay in T47D cells transfected with the HER3 3’UTR or control vector. (PDF 150 kb) [file 12943_2018_862_MOESM9_ESM.pdf]

# Figure S6

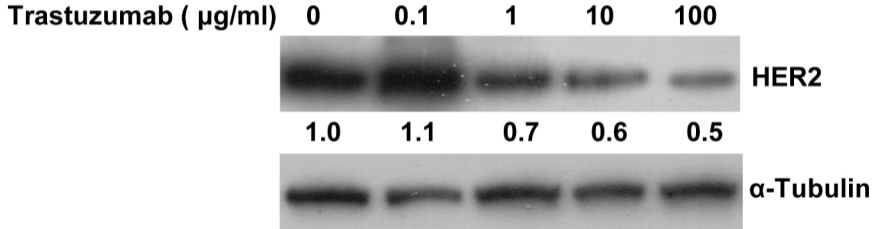

Supplement: Supplementary file 10 — Figure S6. Effect of trastuzumab on HER2 protein levels. Western blot analysis of HER2 levels in AU565 cells treated with the indicated concentrations of trastuzumab. Numbers below the blot indicates quantification shown on Western blot after normalization. (PDF 282 kb) [file 12943_2018_862_MOESM10_ESM.pdf]
